# Successful cardiac resuscitation with extracorporeal membrane oxygenation in the setting of persistent ventricular fibrillation: a case report

**DOI:** 10.1186/1756-0500-7-782

**Published:** 2014-11-04

**Authors:** Mehrdad Golian, Darren Freed, Davinder S Jassal, Amir Ravandi

**Affiliations:** Section of Cardiology, Department of Internal Medicine, Faculty of Medicine, University of Manitoba, 409 Tache Avenue, Winnipeg, Manitoba R2H 2A6 Canada; Section of Cardiac Surgery, Department of Surgery, Faculty of Medicine, University of Manitoba, Winnipeg, Manitoba Canada; Institute of Cardiovascular Sciences, St. Boniface Research Centre, University of Manitoba, Winnipeg, Manitoba Canada; Department of Radiology, Faculty of Medicine, University of Manitoba, Winnipeg, Manitoba Canada

**Keywords:** ECMO, Resuscitation, Percutaneous coronary intervention, Arrhythmia

## Abstract

**Background:**

Extracorporeal membrane oxygenation (ECMO) technology is a viable option for short-term support in the setting of acute cardiac ischemia. To supplement cardiopulmonary resuscitation (CPR) in select patients, ECMO is used successfully for witnessed in hospital cardiac arrest. In the setting of an acute myocardial infarction (MI), bridging to a revascularization procedure is important in improving overall survival.

**Case presentation:**

We describe the first known case of a 56-year-old Caucasian male with an anterior ST elevation myocardial infarction (STEMI) who underwent percutaneous coronary intervention (PCI) in which the entire procedure was carried out with the patient being in persistent ventricular fibrillation (VF) resistant to defibrillation on ECMO support. Subsequent to revascularization, the patient’s cardiac rhythm converted back to sinus rhythm with a single defibrillation shock with excellent neurologic recovery.

**Conclusion:**

Our case highlights the importance of early initiation of ECMO during PCI in achieving both improved cardiac and neurological outcomes during an acute coronary syndrome (ACS).

## Background

Extracorporeal membrane oxygenation (ECMO) technology has advanced significantly and is readily available at the bedside. This is a viable option for short-term support in the setting of acute cardiac ischemia. According to the 2003 USA National Registry of Cardiopulmonary Resuscitation, in hospital cardiac arrest has a poor prognosis with an overall survival to hospital discharge rate of 17% with conventional cardiopulmonary resuscitation (CPR) [1]. One of the most common causes of cardiac arrest is ventricular fibrillation (VF) secondary to ischemia which carries an improved prognosis if successfully defibrillated with survival to hospital discharge of 34% [1]. In cases with refractory ischemic VF, definitive therapy with percutaneous coronary intervention (PCI) may not be possible without anoxic brain injury secondary to hemodynamic collapse.

Cardiopulmonary resuscitation was introduced in the 1960’s as a lifesaving method in patients with cardiac arrest [2]. To supplement CPR in select patients, extracorporeal membrane oxygenation (ECMO) is used successfully for witnessed in hospital cardiac arrest [2]. In the setting of an acute myocardial infarction (MI), bridging to a revascularization procedure is important in improving overall survival.

We describe the first known case of PCI in which the entire procedure was carried out with the patient being in persistent VF resistant to defibrillation on ECMO support. Subsequent to revascularization, the patient’s cardiac rhythm converted back to sinus rhythm with a single defibrillation shock with excellent neurologic recovery (cerebral performance category 1).

## Case presentation

**Figure 1:**
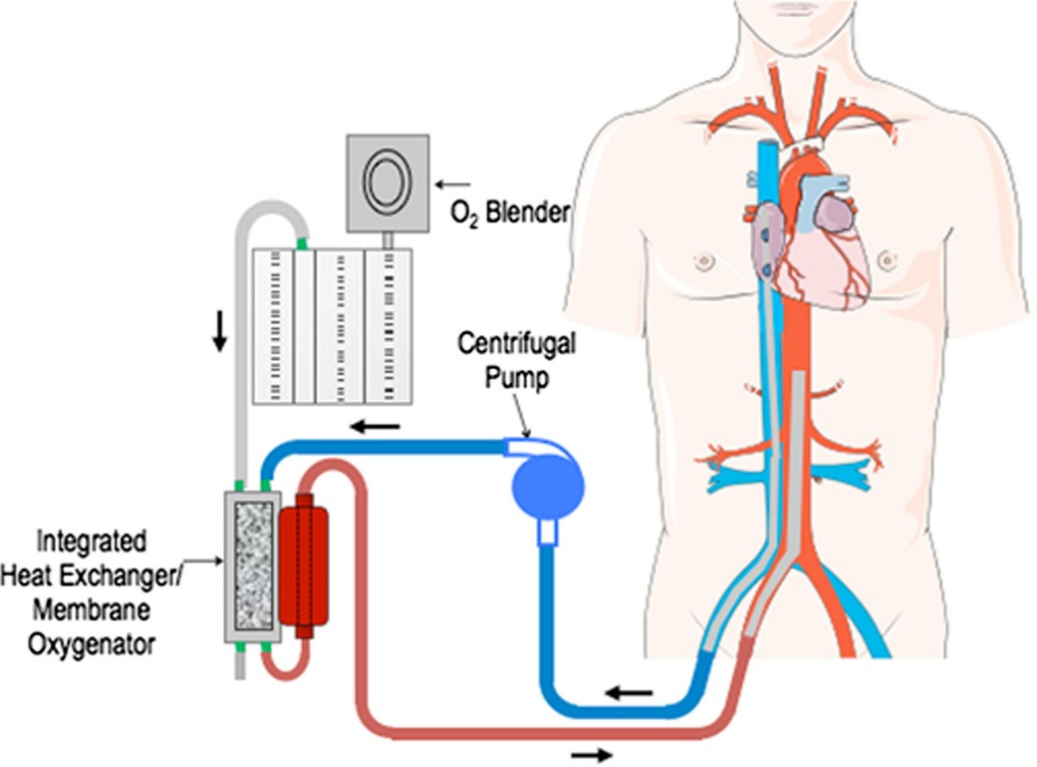
**Schematic diagram demonstrating the circuit for extracorporeal membrane oxygenation support used at our institution.**

A 56-year-old Caucasian male with elevated lipids and smoking history presented to a peripheral hospital with an anterior ST elevation myocardial infarction. The patient developed pulseless ventricular tachycardia (VT) in the peripheral hospital emergency room for which he was successfully defibrillated to normal sinus rhythm after two attempts with 360 J. The total resuscitation period for the first arrest was 15 minutes. Prior to dispatch to the primary PCI center, the patient was started on parenteral amiodarone with a loading dose of 150 mg followed by 50 mg/hr. En route to the tertiary care hospital with PCI capabilities, the patient suffered a second cardiac arrest with pulseless VT/VF. Immediate CPR was initiated by the paramedics prior to arrival at the primary PCI centre. In the ER, the patient required intubation and 40 minutes of CPR with incessant VF refractory to defibrillation and anti-arrhythmic therapy. Specifically, the patient received a total of 4 mg of epinephrine and a total of 7 defibrillation shocks (360 J each). He also received lidocaine 100 mg and amiodarone 150 mg loading doses followed by infusions of both anti-arrhythmic agents. He was started on lidocaine 2 mg/min and continued on the amiodarone 50 mg/hr intravenously. Due to his young age and witnessed arrest, a decision was made to initiate ECMO for hemodynamic support in the emergency department. Peripheral veno-arterial ECMO access was obtained using 17 size French (F) arterial cannula and 24/29 F venous cannula (Cook Medical, Bloomington, IN). A Maquet Rotaflow centrifugal pump and a Quadrox oxygenation system were used to complete the ECMO circuit (Masquet, Rastaat, Germany) as shown in Figure 1. The patient was anti-coagulated with heparin during the ECMO procedure aiming for a PTT of 59–99. The total down time for the second cardiac arrest was 40 minutes until the establishment of ECMO in the cath lab.

**Figure 2:**
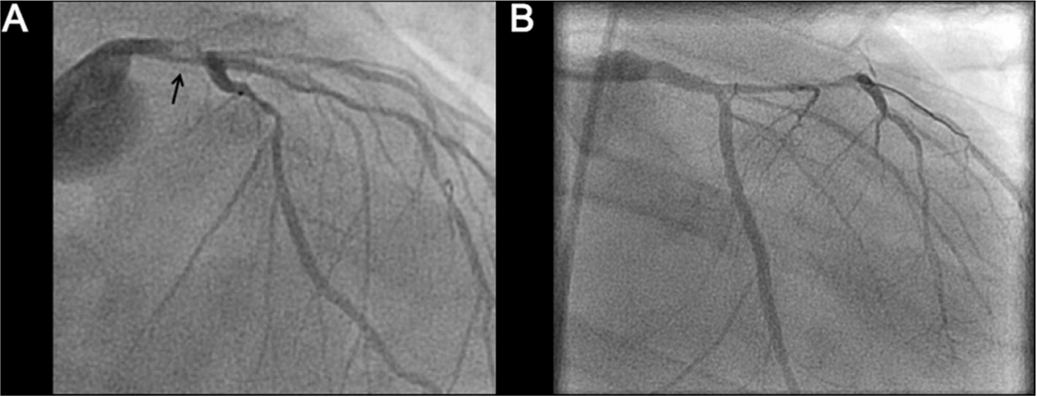
**Coronary angiography prior to and post intervention: (A) Left coronary anatomy prior to percutaneous coronary intervention demonstrating distal left main thrombus (arrow). (B)** Patent left main post coronary intervention with a 3.0 × 8 mm drug eluting stent and post dilated with a 3.5 × 8 non compliant balloon. The obtuse marginal also underwent angioplasty with 2.5 × 38 mm Endeavor drug eluting stent (Medtronic, Minnesota, US) and post dilated with a 2.75 × 15 mm non-compliant balloon.

**Figure 3:**
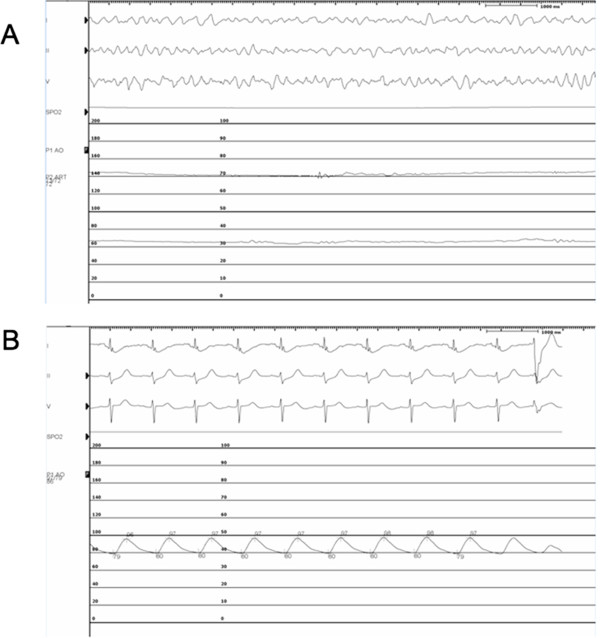
**Intra operative monitor prior to and post intervention: (A) Incessant ventricular fibrillation on venous-arterial extracorporeal membrane oxygenation support with a mean arterial pressure of 72 mmHg. (B)** Post percutaneous coronary intervention demonstrating return to normal sinus rhythm after successful defibrillation.

His initial laboratory investigations demonstrated severe acidosis with a pH of 6.9, lactate of 10 mmol/L, and elevated liver enzymes consistent with shock liver. On ECMO support, the patient was taken for urgent cardiac catheterization which confirmed a 90% thrombotic lesion in the distal left main, 80% disease in the mid left anterior descending artery (LAD), and 90% disease in the first obtuse marginal (OM) with a dominant right coronary artery free of any obstructive lesions (Figure 2A). A 3 mm × 8 mm Xience V (Abbott Vascular, Illinois, US) drug eluting stent (DES) was used for the distal left main, 2.5 × 15 mm Xience DES (Abbott Vacular, Illinois, US) for the mid LAD, and a 2.5 × 38 mm Endeavor DES (Medtronic, Minnesota, US) for the first OM with successful angiographic results (Figure 2B). An attempt to completely revascularize both culprit and non-culprit coronary lesions was performed as the patient was in cardiogenic shock. The patient remained in refractory VF throughout the entire PCI procedure (Figure 3A). Upon complete percutaneous revascularization, a single defibrillation shock restored the patient to normal sinus rhythm after 60 minutes of refractory VF (Figure 3B). The patient received therapeutic hypothermia for neuro-protection in the intensive care unit. After 48 hours post PCI, the patient was taken off ECMO support. Although his initial transthoracic echocardiogram (TTE) revealed a left ventricular ejection fraction (LVEF) of less than 20% with global hypokinesis, at day 9 post PCI, the LVEF improved to greater than 50%. The creatinine kinase and high sensitivity troponin T peaked at 3251 U/L and 4130 ng/L, respectively. The patient was discharged home 10 days later without any neurologic deficits.

## Discussion

Only recently has technology advanced enough to provide bedside mechanical cardiopulmonary support in an emergency situation [3, 4]. Published guidelines from the Extracoporeal Life Support Organization indicate use of ECMO in cases of reversible cardiac arrest or pulmonary failure where conventional therapy has failed and the mortality risk is considered to be greater than 50% [3, 4]. In the case of veno-arterial ECMO, common indications include cardiogenic shock post acute MI, post cardiac surgery unable to wean off cardiopulmonary bypass, myocarditis, bridge to transplant in end stage heart disease, or in the setting of early graft failure post heart transplant [3, 4].

There have been a number of studies evaluating the role of ECMO in the setting of an acute coronary syndrome (ACS) [2, 4–6]. In a 3 year prospective observational study of patients receiving greater than 10 minutes of CPR, there was a higher survival rate to discharge and a better 1-year survival in patients treated with ECMO as compared to conventional CPR [2]. In a retrospective study of 81 patients requiring ECMO for refractory cardiogenic shock, Coombs et al. found that ECMO support could rescue 40% of otherwise fatal refractory cardiogenic shock with good quality of life outcome as measured by mental and physical health [6]. In addition, use of ECMO in combination with intra-arrest PCI was associated with a higher survival rates in patients who were unresponsive to conventional CPR [4–6]. This data supports the use of ECMO in ACS refractory to conventional therapy where there are no contraindications identified as a bridge to complete revascularization.

## Conclusion

Our case highlights the importance of early initiation of ECMO during PCI in achieving both improved cardiac and neurological outcomes during an ACS.

## Consent

Written informed consent was obtained from the patient for publication of this case report and accompanying images. A copy of the written consent is available for review by the Editor-in-Chief of this journal.

## Abbreviations

ACS: Acute coronary syndrome
CPR: Cardiopulmonary resuscitation
DES: Drug eluting stent
ECMO: Extracorporeal membrane oxygenation
LAD: Left anterior descending artery
MI: Myocardial infarction
OM: Obtuse marginal
PCI: Percutaneous coronary intervention
STEMI: ST elevation myocardial infarction
TTE: Transthoracic echocardiogram
VT: Ventricular tachycardia
VF: Ventricular fibrillation.
